# Investigating the Effect of Silver Nanoparticles on the Fluorescence Intensity of Bambuterol and its Active Metabolite Terbutaline Using FRET

**DOI:** 10.1007/s10895-023-03182-7

**Published:** 2023-02-24

**Authors:** Shymaa M. Abd Elhaleem, F. Elsebaei, Sh. Shalan, F. Belal

**Affiliations:** Department of Pharmaceutical Analytical Chemistry, Faculty of Pharmacy, Mansoura University, Mansoura, 35516 Egypt

**Keywords:** Silver nanoparticles, Bambuterol, Terbutaline, Fluorescence quenching, Stern–Volmer equation

## Abstract

Silver nanoparticles (AgNPs) were found to significantly quench the fluorescence of bambuterol hydrochloride (BAM) and its active metabolite terbutaline sulfate (TER). The intrinsic fluorescence intensity of each of BAM (at 264/292 nm) and TER (at 276/306 nm) decreased by the gradual addition of AgNPs. Quenching of the steady state fluorescence of BAM and TER probably resulted from the energy transfer to the photo-excited state of AgNPs. The estimated Stern–Volmer quenching constant at several temperature settings proved that the quenching mechanism of the two drugs was dynamic quenching in case of BAM while it was static quenching in case of TER. The number of binding sites, binding constants, and corresponding thermodynamic parameters depending on the interaction system were estimated at 293, 313, and 333 °K and the results obtained were interpreted.

**Supplementary Information:**

The online version contains supplementary material available at 10.1007/s10895-023-03182-7.

## Introduction

Metal nanoparticles (NPs) have pulled considerable interest of researchers because of their remarkable size dependent optoelectronic properties [1]. In this regard, efforts have been dedicated to the synthesis and characterization of metal nanoparticles. Gold, silver and copper NPs display obvious electronic absorption bands in the visible region of the spectrum because of surface plasmon excitation [2]. The most widely used are AgNPs due to their optical, catalytic, and electrical features [3–6]. The optical properties of AgNPs are because they exhibit shining colors as a result of absorption of surface plasmon resonance (SPR), which is really because of combination of light absorption and scatter [7]. The optical features of a fluorophore found near the metal NPs are influenced by the near-field electro dynamical environment [3]. The interaction of fluorescent molecules with metal NPs can lead to fluorescence quenching or enhancement relying on the distance between the molecule and metal surface. In presence of fluorophore in very short distances near the surface of the metal, non-radiative energy is transferred to surface plasmon in the metal, thus enhancing the performance of metal nanoparticles as energy acceptors [8]. Considering the distance dependence of the energy transfer process, Förster Resonance Energy Transfer (FRET) are ascribed to take place [9]. Valuable information is provided on the nature of interaction between the fluorescent molecule and the quenching agent according to Stern–Volmer equation. There are two types of fluorescence quenching processes. First type is dynamic (collisional) quenching which results from collision between fluorophore and quenching agent. The dynamic quenching offers a non-radiative way for loss of the excited state energy. Static quenching is the second type of quenching, in which a non-fluorescent complex is formed [10].

**Fig. 1:**
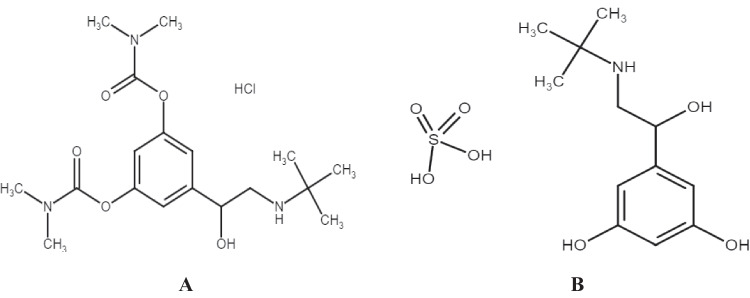
Chemical structures of **A** bambuterol hydrochloride (BAM) and **B** terbutaline sulfate (TER)

Bambuterol is: (RS)-5-(2-tert-Butylamino-1-hydroxyethyl)-m-phenylene bis(dimethylcarbamate). (Fig. 1A). It is an inactive pro-drug for terbutaline. A long-acting beta-_**2**_ adreno-receptor agonist employed as a bronchodilator in the treatment of asthma. Terbutaline [2-*tert*-Butylamino-1-(3,5-dihydroxyphenyl) ethanol] (Fig. 1B) is a direct-acting sympathomimetic drug with a selective action on β_2_ receptor (a β_2_ agonist). Terbutaline is formulated and given as sulfate salt for its broncho-dilating properties in reversible airways obstruction, as occurs in asthma and in some patients with chronic obstructive pulmonary diseases (COPD). It also decreases contractility of the uterus [11].

The influence of AgNPs on the fluorescence of either BAM or TER has not been reported yet. In this paper, we aimed to study the energy transfer and the fluorescence behavior of both BAM and TER with AgNPs. Similar studies have been conducted on some compounds, viz ciprofloxacin [12], norfloxacin [13],bovine serum albumin [14], acridine orange [15], chlorophyll [16], coumarine dye [17] and amino acids [18].

## Experimental

### Chemicals

- Silver nitrate (AgNO_3_), sodium borohydride (NaBH_4_), and polyvinyl pyrrolidone (PVP) were obtained from Sigma-Aldrich (USA).
- Bambuterol hydrochloride was supplied by Chemipharm Pharmaceutical Co., Cairo, Egypt.
- Terbutaline sulfate was obtained as a gift from Sedico Pharmaceutical Co., Cairo, Egypt.
- Ultrapure water was used throughout the study and all chemicals were of Analytical Grade.

### Apparatus

- The fluorescence spectra were recorded on Cary Eclipse Spectrofluorometer equipped with Xenon flash lamp. The study was conducted at 800 V, smoothing factor of 20, and the emission slit width was 5 nm.
- Magnetic stirrer (Product of Daihan Scientific Co, ltd, South Korea).
- High Resolution Transmission Electron Microscopy (HR-TEM) images were obtained on JSM-2100 (JEOL, Tokyo, Japan) with an accelerating voltage of 200 kV and using 200 mesh carbon coated Cu-grid.

### Synthesis and Characterization of Silver Nanoparticles

Colloidal solution of AgNPs (6.0 × 10 ^−5^ M) was prepared adopting a previously reported chemical reduction method [19]. It involved drop-wise addition of 10 mL of AgNO_3_ solution (0.01 M) to 30 mL of NaBH_**4**_ solution (0.02 M) that had been chilled in an ice bath. Vigorous stirring was performed until obtaining light yellow solution indicating full reduction of silver ions. A capping agent, PVP (0.3%w/v) was further added in order to stabilize the produced nanoparticles. The PVP capped AgNPs were finally observed and investigated by both UV–visible spectroscopy and HR-TEM. UV-spectrum of AgNPs showed an intense absorption maximum at 385 nm, as presented in Fig. S1. HR-TEM micrograph indicated spherical monodisperse particles with sizes of $14 \pm 2\,nm,$ as illustrated in Fig. S2.

### Quantum Yield

The fluorescence quantum yield (QY) of both BAM and TER was determined with phenol (Ph) in hexane as a standard (QY: 0.075 at 270 nm) [20] according to the Eq. (1) [21]:

1 $${{\varvec{\phi}}}_{{\varvec{X}}}={{\varvec{\phi}}}_{{\varvec{P}}{\varvec{h}}}\times \left[\frac{{{\varvec{F}}}_{{\varvec{X}}}}{{{\varvec{F}}}_{{\varvec{P}}{\varvec{h}}}}\right]\times \left[\frac{{{\varvec{A}}}_{{\varvec{P}}{\varvec{h}}}}{{{\varvec{A}}}_{{\varvec{X}}}}\right]\times {\left[\frac{{\upeta }_{{\varvec{X}}}}{{\upeta }_{{\varvec{P}}{\varvec{h}}}}\right]\boldsymbol{ }}^{2}$$

where:

${\varvec{\phi}}$ refers to the quantum yield**,**

**F** represents the measured integrated fluorescence emission intensity,

**A** stands for the absorbance value of the solution,

and **η** is the refractive index of the solvent used.

The subscripts **Ph** and **X** denote phenol and BAM/TER respectively. It was found that the quantum yield values are 0.067 for BAM and 0.138 for TER.

### Standard Solutions

Stock standard solutions of each of BAM and TER (100.0 μg/mL) were prepared in two 100-mL volumetric flasks by dissolving 0.01 gm of each drug in 60 mL of ultra-pure water, sonicating for 10 min, and then completing to volume with the same solvent. All drug solutions were light-protected and stored in a refrigerator. Proper dilutions of the stock solutions were done as appropriate with ultra-pure water to obtain working solutions.

### Measurements

To examine the impact of AgNPs on the fluorescence behavior of BAM, a series of 3.0 μg/mL BAM sample solutions with different concentrations of AgNPs (0, 0.06; 0.12; 0.18; 0.3; 0.42; 0.48; 0.6; 0.9; 1.2; 1.8; 2.4 and 3.0 µM) were prepared in 10 mL volumetric flasks. To investigate the influence of AgNPs on the fluorescence intensity of TER, a series of 1.0 μg/mL TER sample solutions with different concentration of AgNPs (0; 0.06; 0.12; 0.3; 0.42; 0.48; 0.6; 0.9; 1.2; 1.8; and 2.4 µM) were prepared in 10 mL calibrated flasks. All samples were spectrofluorimetrically measured at 264/292 nm and 276/306 nm for BAM and TER, respectively.

## Results and Discussion

### Effects of AgNPs on Fluorescence Properties of BAM and TER

**Fig. 2:**
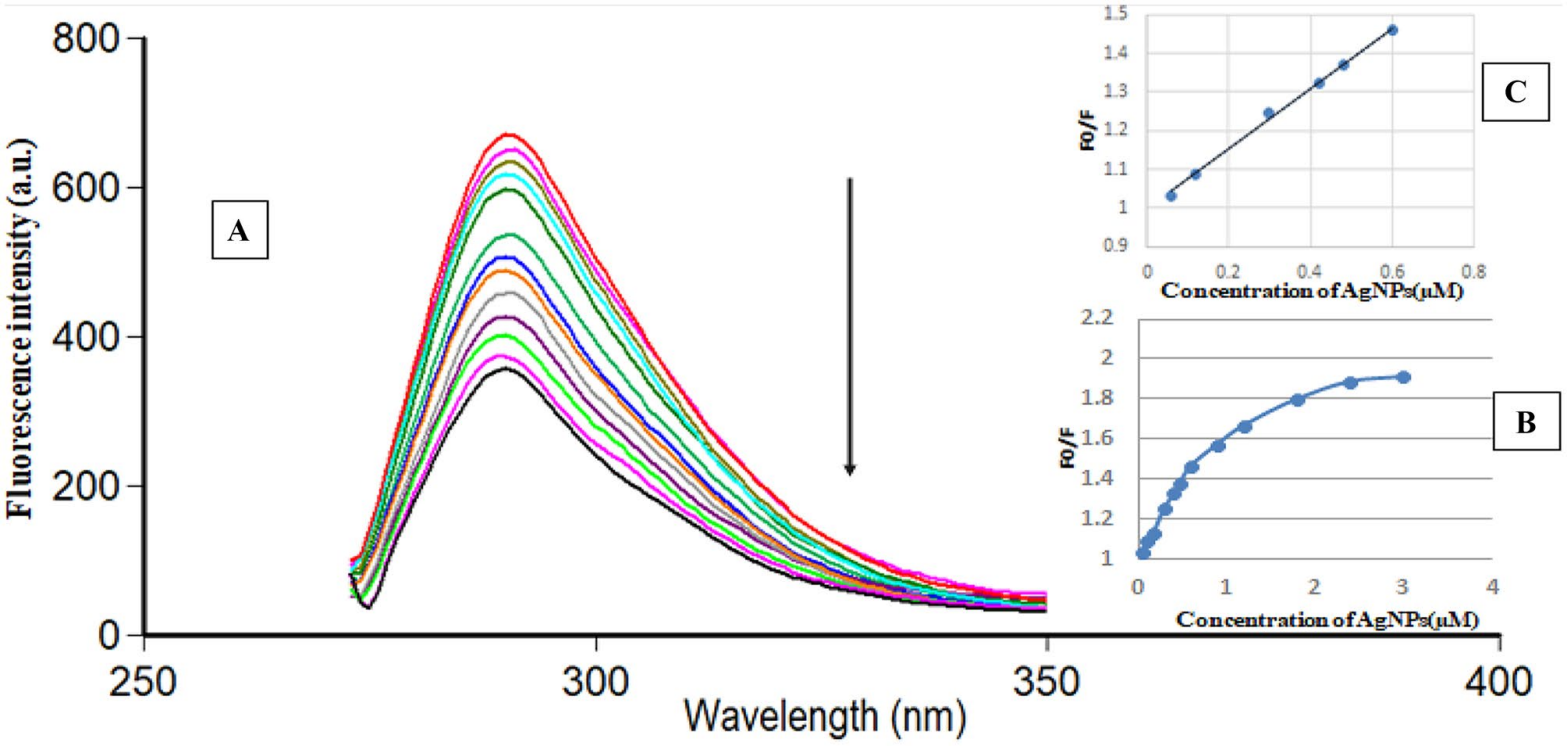
**A**: Fluorescence emission spectra of BAM 3.0 μg/mL after addition of increasing concentrations of AgNPs from top to bottom: 0.0 μM, 0.06 μM, 0.12 μM, 0.18 μM, 0.3 μM, 0.42 μM,0.48 μM, 0.6 μM, 0.9 µM, 1.2 µM, 1.8 µM, 2.4 µM and 3.0 µM. **B**: Plot of F_0_/F versus concentrations of AgNPs (µM) and **C**: the plot showing the linearity fitting over narrow concentration ranges of the corresponding curve

BAM and TER are fluorescent drugs [22, 23]. Figure S3 shows the excitation and emission spectra of BAM at 264 nm and 292 nm, respectively and after addition of AgNPs, there was a significant quenching of its fluorescence. With increasing AgNPs concentration, the fluorescence quenching increased, suggesting the formation of BAM-AgNPs complex [13], as illustrated in Fig. 2A. Plots of F_0_/F versus concentration of the prepared AgNPs [µM] exhibited exponential growth over the concentration ranges of 0.06–3.0 µM (Fig. 2B), while a linear relationship was found within the range of 0.06–0.6 µM with R^2^ of 0.9969 (Fig. 2C).

**Fig. 3:**
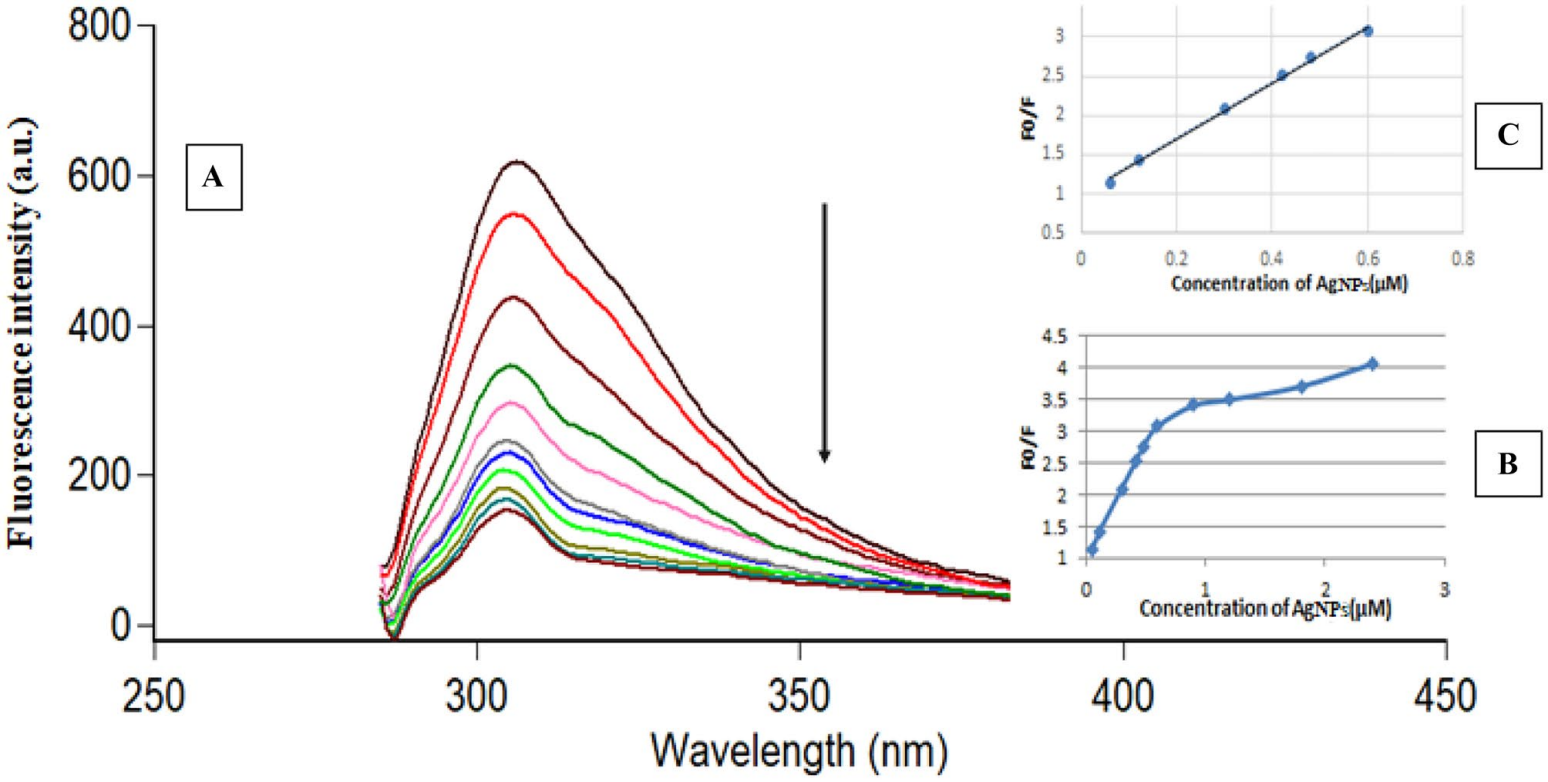
**A**: Fluorescence emission spectra of (TER) 1.0 μg/mL after addition of increasing concentrations of AgNPs from top to bottom: 0.0 μM, 0.06 μM, 0.12 μM, 0.3 μM, 0.42 μM,0.48 μM, 0.6 μM, 0.9 µM, 1.2 µM, 1.8 µM and 2.4 µM. **B**: Plot of F_0_/F versus concentrations of AgNPs (µM) and **C**:the plot showing the linearity fitting over narrow concentration ranges of the corresponding curve

For TER, the excitation and emission peaks were at 276 nm and 306 nm, respectively. It is interesting to see the remarkable quenching of TER fluorescence upon gradual addition of AgNPs even in the micromolar concentration range, as demonstrated in Fig. S4. The higher the concentration of AgNPs added to TER, the greater the fluorescent quenching that would occur, as illustrated in Fig. 3A. Plots of F_0_/F versus concentration of PVP capped AgNPs [µM] indicated exponential growth over the concentration range of 0.06–2.4 µM (Fig. 3B), while a linear relationship is noticed in the range of 0.06–0.6 µM with R^2^ of 0.9963 (Fig. 3C).

### Förster Resonance Energy Transfer (FRET)

FRET is a physical non-radiative process of energy transfer between a pair of light-sensitive molecules where energy is transmitted rapidly from a donor molecule (BAM or TER) to an adjacent molecule (acceptor, AgNPs) by means of intermolecular dipole–dipole interactions. Energy preservation necessitates that, the energy gap between the ground states and excited states of donor and acceptor molecules are almost the same [24, 25]. There is a partially spectral overlap between the acceptor absorption spectrum of AgNPs and the emission spectra of the donors (BAM and TER) with λ_em_ 292 nm and 306 nm, respectively (Fig. S5). The much more overlap, the higher efficacy of energy transfer. Concerning FRET, the energy transfer efficiency, E is mainly based on the critical energy transfer distance (R_0_) and the distance (r) between acceptor and donor. It is described by the expression:

2 $$E=1-\frac{F}{\mathrm{F}0}=\frac{{R}_{0}^{6}}{{R}_{0}^{6}+{r}^{6}}$$

where:

F_0_ and F are the fluorescence intensities of BAM and TER in absence and presence of the acceptor (AgNPs), R_0_ is a characteristic distance, called the critical donor–acceptor distance or Förster distance when energy transfer efficiency is 50%, and *r* the distance between acceptor and donor. The value of R_0_ is estimated using the following Eq. (3):

3 $${R}_{0}=0.211{[{K}^{2}{N}^{-4}\Phi\,\mathrm{J}]}^\frac{1}{6}$$

where

K^2^ is the factor expressing the relative orientation of the donor to the acceptor molecule,

*Ф* is the fluorescence quantum yield of the donor in absence of the acceptor,

N is the refractive index for the medium (1.333 for water),

J is the overlap integral between the absorbance spectrum of acceptor and the emission spectrum of donor.

*J* can be easily calculated using the following Eq. (4)

4 $$J=\frac{\sum F(\uplambda )\upvarepsilon (\uplambda ){\uplambda }^{4}\mathrm{\Delta \lambda }}{\sum F(\uplambda )\mathrm{\Delta \lambda }}$$

where F(λ) is the normalized emission intensity of donor for a particular wavelength λ, ε(λ) is the acceptor molar extinction coefficient at wavelength λ in unit of cm^−1^ mol^−1^ L [26].

From previous equations, *E* and *J* can be simply calculated; thus, R_0_ and r can be determined. The overlap of the emission spectra of BAM and TER with the absorption spectrum of AgNPs is shown in Fig. S5. The value of *J* could be calculated by means of integrating the spectrum presented in Fig. S5, and *E* was determined to be 0.3151 for BAM and 0.6752 for TER. In this study N = 1.333 [27], K^2^ = 2/3, and *Ф* = 0.067 for BAM and 0.138 for TER were determined. The values of R_0_ for BAM and TER were found to be 2.582 and 2.479 Å, respectively, while the values of r for BAM and TER were further found to be 2.939 and 2.194 Å, respectively. The distance between AgNPs and BAM or TER is 2.939 or 2.194 Å, which is much lower than 70 Å [28] pointing out that the energy transferred from BAM or TER to AgNPs with high probability.

### Elucidation of Fluorescence Quenching

The fluorescence quenching of each of BAM and TER by AgNPs was found to be concentration-dependent. The process of FRET (assuming that the excited BAM or TER with AgNPs formed as 1:1 complex) can be expressed as follows

5 $$\mathrm{BAM}/\mathrm{TER}\xrightarrow{\mathrm{hv}}{\mathrm{BAM}}^{*}/{\mathrm{TER}}^{*}$$

6 $${\mathrm{BAM}}^{*}/{\mathrm{TER}}^{*}+\mathrm{Ag}\xrightarrow{\mathrm{k}}[\mathrm{BAM}-\mathrm{Ag}]/[\mathrm{TER}-\mathrm{Ag}]$$

7 $${\mathrm{BAM}}^{*}/{\mathrm{TER}}^{*}\longrightarrow\mathrm{hv}+\mathrm{BAM}/\mathrm{TER}$$

Equation (5) represents excitation of BAM and TER by absorption of photons. Equation (6) represents non-radiative, de-excitation process, which is FRET process between the two drugs and AgNPs. Equation (7) represents radiative de-excitation process which can be also known as fluorescence emission. K is the quenching constant that can be determined from the linear relation of Stern–Volmer equation. In order to explore the quenching type of the fluorescence of BAM and TER, the Stern Volmer equation [29] was applied at different temperature settings [ 293, 313 and 333 °K] adopting the following Eq. (8):

8 $${\mathrm{F}}_{0}/\mathrm{F}=1+{\mathrm{K}}_{\mathrm{sv}} [Q]$$

[Q] stands for the concentration of quencher AgNPs.

K_sv_ is Stern–Volmer quenching constant.

**Fig. 4:**
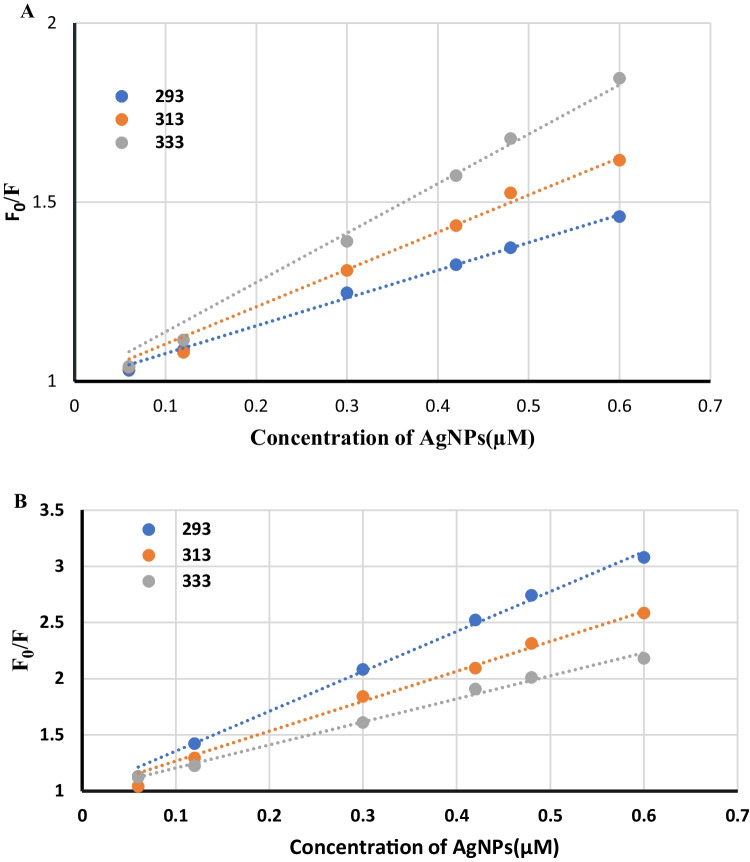
Stern–volmer plots of BAM (**A**) and TER (**B**) at 293, 313 and 333 °K

The values of F_0_/F against concentration of AgNPs and linear relation are shown in Stern Volmer plots (Fig. 4).

The obtained quenching constant values were determined at three different temperature settings, as illustrated in Table 1. For BAM, the values of the Stern Volmer constants increase with increasing temperature, pointing out that the type of fluorescence quenching is dynamic (collisional) quenching. While for TER, the quenching is static as the values of K_sv_ decreases with increasing temperature.

**Table 1:** Application of Stern–volmer and modified Stern–Volmer equations for interaction of BAM and TER with AgNPs at different temperatures

	**Stern–Volmer equation**				**Modified Stern–Volmer equation**		
**Drug**	**Temperature (K)**	**K**_**SV**_ **(L.mol**^**−1**^**)**	**kq** **(L.mol**^**−1**^** S**^**−1**^**)**	**R**^**2**^	**Binding constant (K, L mol**^**−1**^**)**	**No of binding sites**	**R**^**2**^
**BAM**	**293**	7.75 $\times$ 10^5^	7.75 $\times$ 10^13^	0.9969	1.4 $\times$ 10^7^	1.1976	0.9949
	**313**	1.04 $\times$ 10^6^	1.04 $\times$ 10^14^	0.995	2.84 $\times$ 10^7^	1.2258	0.9972
	**333**	1.38 $\times$ 10^6^	1.38 $\times$ 10^14^	0.9994	1.8 $2\times$ 10^8^	1.3341	0.9979
**TER**	**293**	3.55 $\times$ 10^6^	3.55 $\times$ 10^14^	0.9963	$3.76\times$ 10^6^	1.0036	0.9989
	**313**	2.66 $\times$ 10^6^	2.66 $\times$ 10^14^	0.9938	5.80 $\times$ 10^6^	1.0528	0.9961
	**333**	$2.05\times$ 10^6^	2.05 $\times$ 10^14^	0.9939	$4.61\times$ 10^6^	1.0551	0.9960

9 $${\mathrm{K}}_{\mathrm{sv}}={\mathrm{k}}_{\mathrm{q}}\times {\tau }_{0}$$

Equation (9) represents bimolecular quenching, where k_q_ is the bimolecular quenching rate constant and τ_0_ (10^−8^ s) is the fluorescence lifetime of the substance without AgNPs [12, 30].The values of k_q_ were calculated and abridged in Table 1. Upon concluding the mechanism of quenching, it was valuable to determine the degree of association between each of BAM and TER with AgNPs. Hence, Modified Stern–Volmer Eq. (10) was applied to calculate (n) number of binding sites and (K) the binding constant [31].

10 $$\mathrm{log}\frac{\mathrm{F}0-F}{F}=\mathrm{log}\;K+n\;\mathrm{log}\;Q$$

**Fig. 5:**
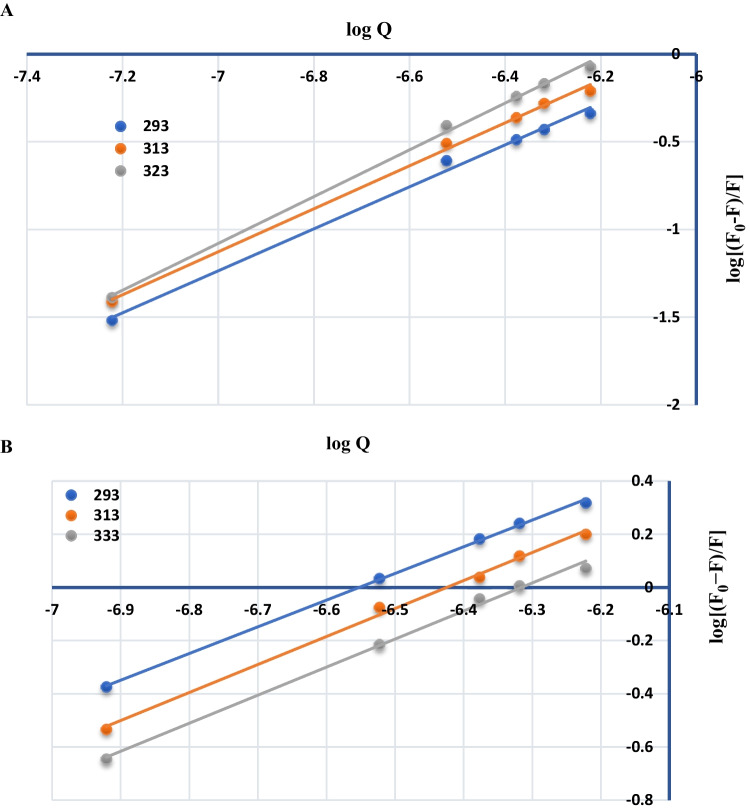
Plot between log (F_0_-F)/F vs. log Q for BAM (**A**) and TER (**B**)

The relationships between log (F_0_-F)/F and log Q at different temperature settings were demonstrated in Fig. 5, a linear relationship with intercept corresponding to log K and slope corresponding to n were obtained. The values of K and n for both of BAM and TER with AgNPs at various temperatures are abridged in Table 1.

The thermodynamic parameters including the entropy change (ΔS), enthalpy change (ΔH), and free energy change (ΔG^0^) for each of BAM-AgNPs and TER-AgNPs complexes were assessed to determine the force type. These parameters were calculated by Van^'^t Hoff and Gibbs Helmholtz Eqs. (11,12) respectively.

11 $$\mathrm{ln}\;K=\frac{-\mathrm{\Delta H}^\circ }{RT}+\frac{\Delta S^\circ }{R}$$

12 $$\Delta {G}^{0}=\mathrm{\Delta H}^\circ -T\Delta S^\circ$$

where:

K is binding constant,

T is the temperature in Kelvin, and

R is the universal gas constant.

**Fig. 6:**
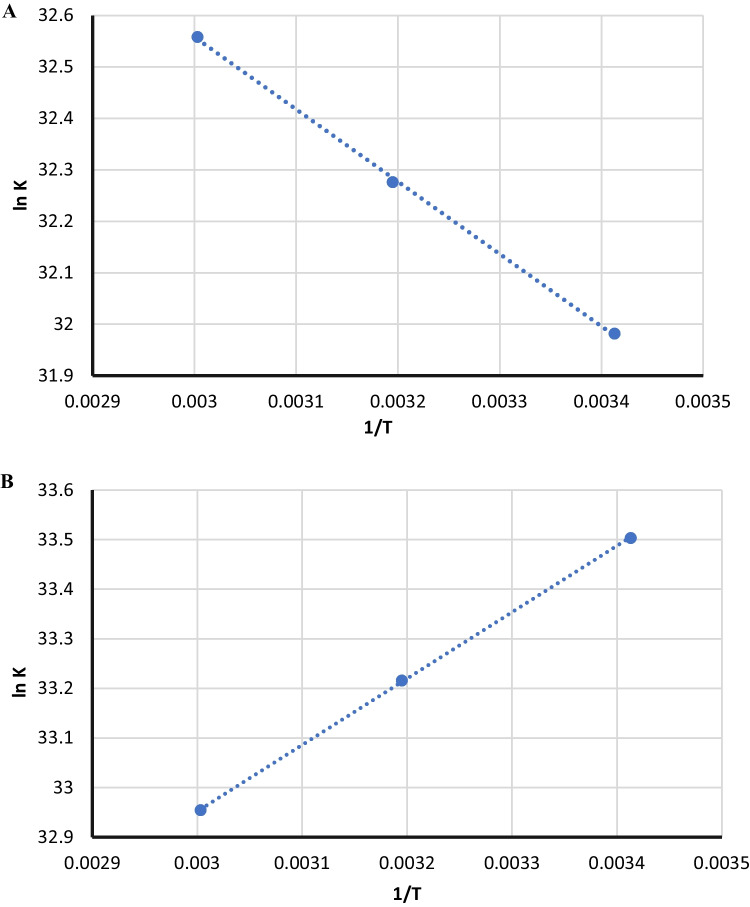
Van't Hoff plot for the BAM-AgNPs (**A**) and TER-AgNPs (**B**) binding interaction

The values of Δ*S°*, ΔH°, and ΔG^0^ were calculated from the relation between ln K and 1/T, as indicated in Fig. 6.

Types of interaction that may occur in complex formation can be categorized into two types of hydrophobic interaction and electrostatic forces. In conditions of electrostatic forces Δ*S° is* above zero *and* ΔH° must be less than zero. In conditions of hydrophobic forces, in turn, (ΔH°, ΔS° > 0), and for both Van der Waals' interactions and H- bonding (Δ*H°*, Δ*S°* < 0) [32]. It was deduced that the dominant binding mechanism for BAM-AgNPs is hydrophobic forces;while for TER-AgNPs, it is electrostatic forces, as shown in Table 2.

**Table 2:** Thermodynamic parameters for BAM-AgNPs and TER-AgNPs mixtures at various temperatures

**Drug**	**Temperature (K)**	**Δ*H*** **kJ/mol**	**Δ*G*** **kJ/mol**	**Δ*S*** **kJ/mol**	**R**^**2**^
**BAM**	293	11 .69	-79.14	0.31	0.9994
	313		-85.34		
	333		-91.54		
**TER**	293	-11.13	-81.45	0.24	0.9999
	313		-86.25		
	333		-91.05		

The difference in mechanism of binding of AgNPs with each of the drug and its metabolite is attributed to the difference in their chemical structure. The metabolite (TER) is more polar with free OH groups available for Hydrogen bonding leading to static forces. BAM, on the other hand, is more bulky and less polar, hence it is more legible for hydrophobic interaction.

## Conclusion

The interaction of AgNPs with each of BAM and TER were investigated. AgNPs were found to quench the fluorescence of both BAM and TER due to two reasons. First; the FRET between donor fluorophore and acceptor molecule. Second reason; the collision between fluorophore and quenching agent. With the addition of increasing concentrations of AgNPs, the intensity of the fluorescence of BAM and TER decreased correspondingly. Stern Volmer quenching constants were calculated at increasing temperature, thus, the quenching mechanism was deduced to be a dynamic (collisional) quenching for BAM and static quenching for TER.

### Supplementary Information

Below is the link to the electronic supplementary material.

Supplementary file1 (DOCX 467 KB)

## Data Availability

All the data and the materials are available all-over the study.
