# Supplementary material for: Investigating the Effect of Silver Nanoparticles on the Fluorescence Intensity of Bambuterol and its Active Metabolite Terbutaline Using FRET
Source: J Fluoresc. 2023 Feb 24;33(5):1717–25. doi: 10.1007/s10895-023-03182-7 (PMC10539440; doi:10.1007/s10895-023-03182-7)
Supplement: Supplementary file 1 — Supplementary file1 (DOCX 467 KB) [file 10895_2023_3182_MOESM1_ESM.docx]

**Fig.S1:** UV-visible spectrum of the prepared spherical AgNPs.

**Fig.S2**: HR-TEM images of AgNPs under different magnifications.

**Fig.S3**: a, a`: Excitation and emission spectra of BAM (3.0 μg/mL) in ultrapure water and b, b`: excitation and emission spectra of BAM (3.0 μg/mL) after addition of

- 1. µmol/L AgNPs.

**Fig.S4:** Excitation and emission spectra of TER (1.0 μg/mL) in ultrapure water (a, a`)

and after addition of 0.18 µmol/L AgNPs (b, b`).

**Fig.S5:** Spectral overlap of emission of BAM (a), TER (b), and absorption of AgNPs (c).

**
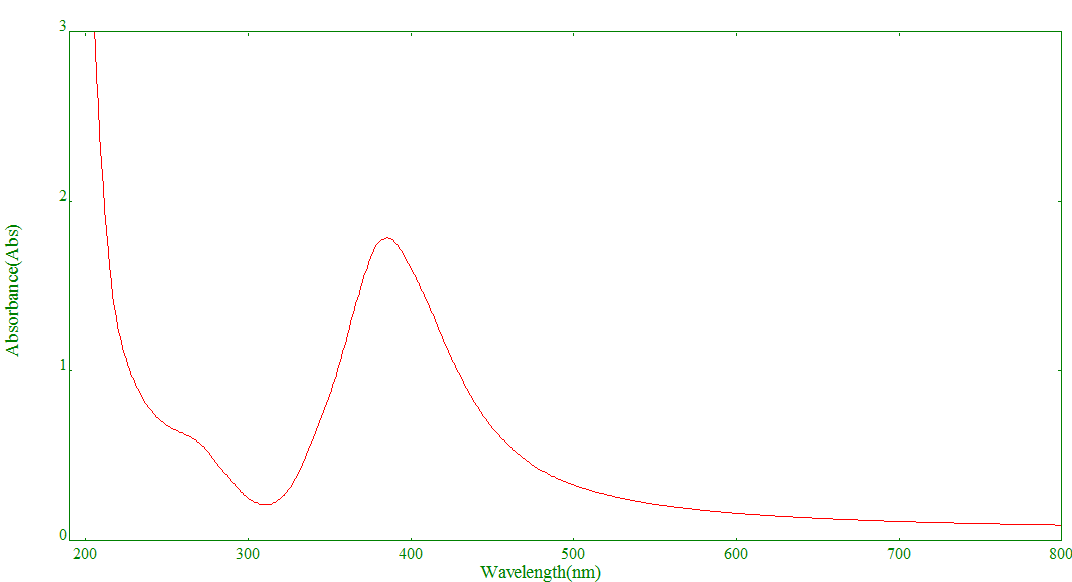
**

**Fig.S1**

| 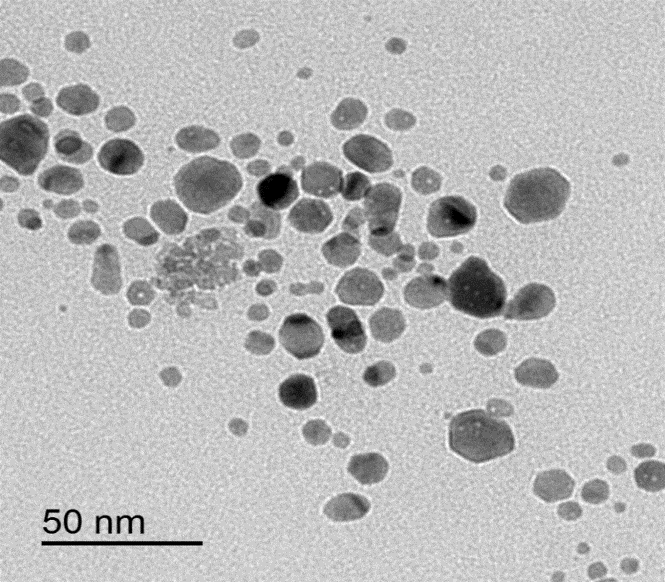 | 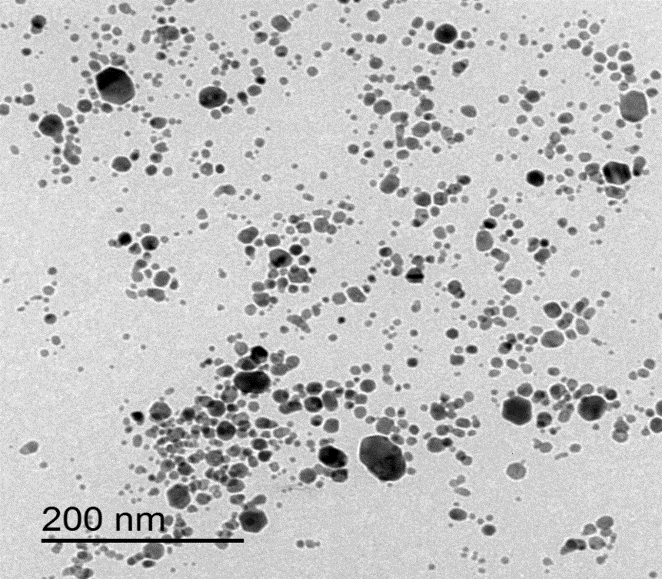 |
| --- | --- |

**Fig.S2**


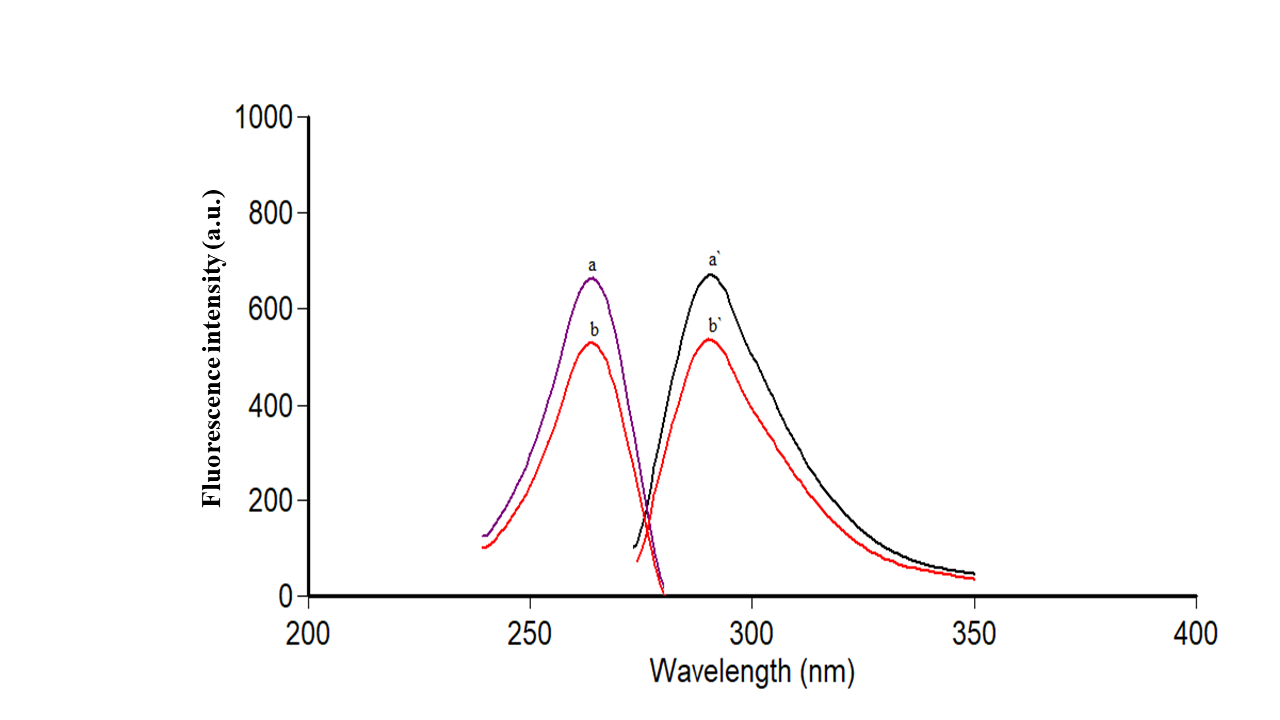


**Fig.S3**


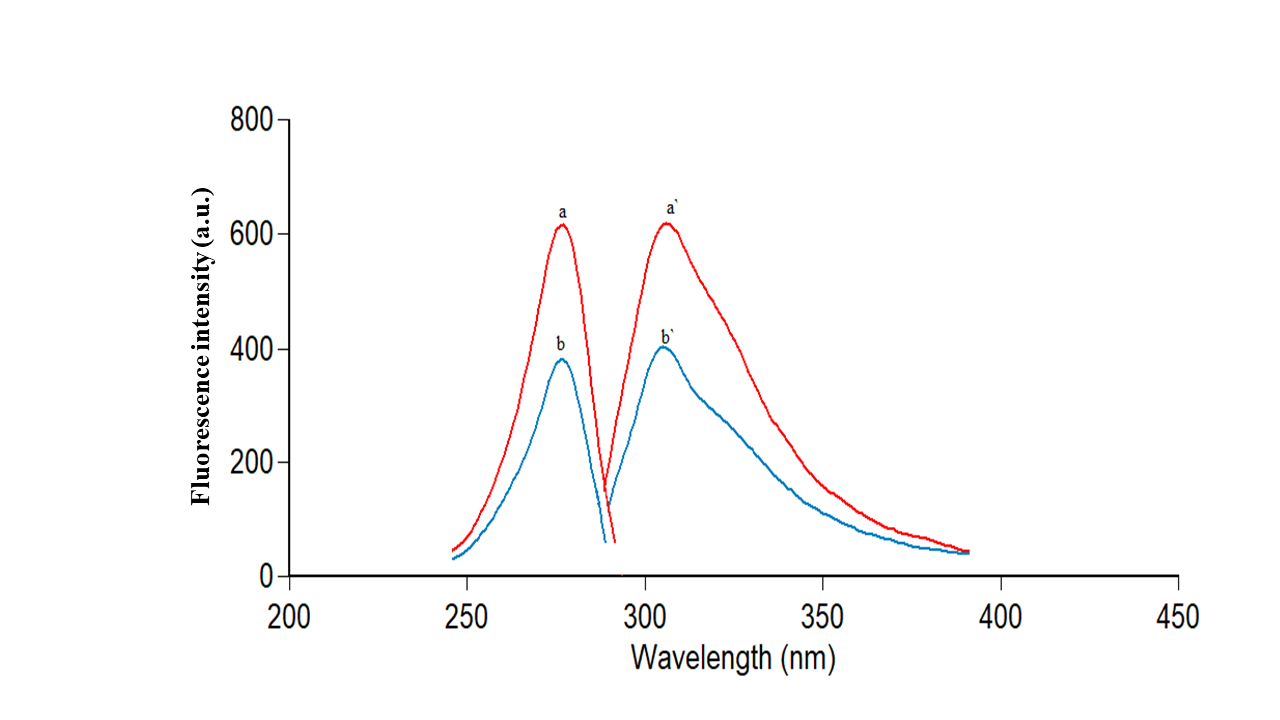


**Fig.S4**


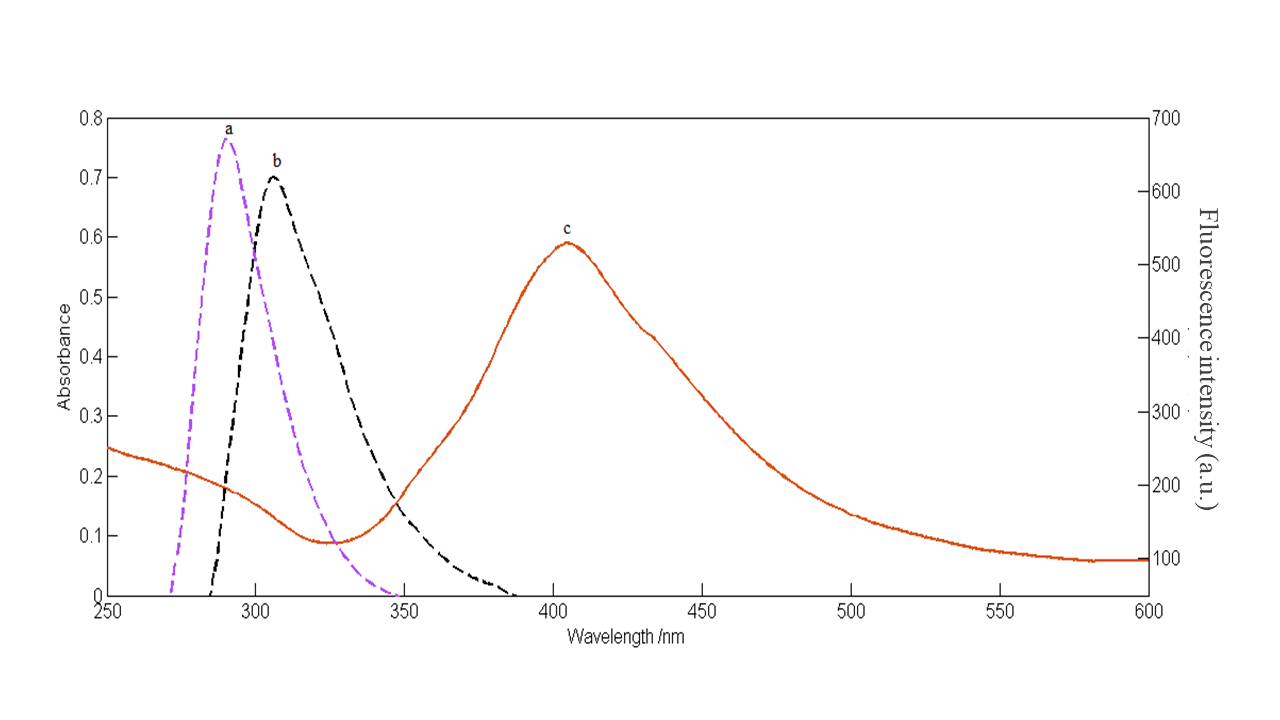


**Fig.S5**
